# Anaemia and its associated factors among diabetes mellitus patients in Ethiopia: A systematic review and meta‐analysis

**DOI:** 10.1002/edm2.260

**Published:** 2021-05-14

**Authors:** Tiruneh Adane, Solomon Getawa

**Affiliations:** ^1^ Department of Hematology and Immunohematology School of Biomedical and Laboratory Sciences College of Medicine and Health Sciences University of Gondar Gondar Ethiopia

**Keywords:** anaemia, diabetes mellitus, Ethiopia, meta‐analysis, systematic review

## Abstract

**Introduction:**

Anaemia is common but often overlooked in diabetes mellitus (DM) patients. There is also no official nationwide survey registry that estimated the prevalence of anaemia in DM patients in Ethiopia. Therefore, the main aim of this study is to determine the countrywide pooled prevalence and associated factors of anaemia in DM patients.

**Methods:**

This systematic review and meta‐analysis were conducted as per the Preferred Reporting Items for Systematic Reviews and Meta‐Analyses (PRISMA) guidelines. STATA 11 software was used for all statistical analysis. Random effects model was used to estimate the pooled prevalence of anaemia and associated factors at a 95% confidence interval (CI) with its respective odds ratio (OR). Subgroup analysis and egger test were used to determine heterogeneity and publication bias, respectively.

**Results:**

Nine articles were included in this systematic review and meta‐analysis with a total of 2889 DM patients. The pooled prevalence of anaemia among DM patients in Ethiopia was 22.11% (95% CI: 15.83–28.39) *I* ^2^ = 94.8%. The prevalence of anaemia in type I and type II DM patients was (16.78% [95% CI: 11.53–22.04]) and (31.12% [95% CI; 9.66–52.58]), respectively. The prevalence of anaemia was higher among male (36.72% [95% CI: 22.58–50.87] *I* ^2^ = 97.6%) than female (27.51% [95% CI: 16.12–38.90] *I* ^2^ = 96.3%). Moreover, the odds of anaemia were higher among patients with age ˃ 60 (OR = 2.98; 95% CI: 1.83, 4.87), low estimated glomerular filtration rate (eGFR) (OR = 8.59; 95% CI: 4.76, 15.57), and duration of illness ≥5 years (OR = 2.66; 95%: 1.38, 5.13).

**Conclusions:**

The result of this review implies that anaemia is a moderate public health problem among DM patients in Ethiopia. Older age, poor glycemic control, low eGFR and longer duration of illness were found to be the contributing factors for the development of anaemia in DM patients. Therefore, by considering the negative impact of anaemia, it is important to include anaemia screening into routine assessment of DM‐related complications targeting patients with older age, poor glycemic control, low eGFR, and longer duration of illness to reduce the magnitude of the problem.

## Graphical abstract

The result of this review implies that anaemia is a moderate public health problem among DM patients in Ethiopia. Therefore, by considering the negative impact of anaemia, it seems important to include anaemia screening into routine assessment of DM‐related complications targeting patients with older age, poor glycaemic control, low eGFR, and longer duration of illness to reduce the magnitude of the problem.

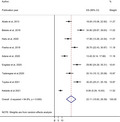

## 1 BACKGROUND

Diabetes mellitus (DM) is a condition primarily defined by the level of hyperglycaemia giving rise to the risk of microvascular damage (retinopathy, nephropathy and neuropathy). It is associated with reduced life expectancy, significant morbidity, and diminished quality of life.^1^ In 2019, 501 million people were estimated to be living with DM in Africa, and this is projected to increase to 704 million by 2030. Ethiopia ranked among the top five countries for the number of people with DM (20–79 years) in this report.^2^ According to a systematic review and meta‐analysis conducted by Nshisso et al.,^3^ 6.5% of Ethiopian adult populations live with DM. The metabolic deregulation associated with DM causes secondary pathophysiologic changes in multiple organ systems.^4^

Haematological changes in red blood cells (RBCs), white blood cells (WBCs), and the coagulation factors are shown to be directly associated with DM.^5^, ^6^ Chronic hyperglycaemia, hyperosmolarity, and increased levels of advanced glycation end‐products affect the RBCs.^7^ Anaemia is a common haematological finding in DM patients.^8^ It is an important global public health problem, affecting the lives of more than 2 billion people globally, accounting for about 30% of the world's population. In Ethiopia, anaemia affects 17% of women and 11% of men aged 15–49 years.^9^

Systemic inflammation, inhibition of erythropoietin (Epo) release, damage to the renal interstitium, efferent sympathetic denervation of the kidney, loss of appropriate Epo, drugs, altered iron metabolism, and hyperglycaemia are some of the factors suggested as the reason for the earlier onset of anaemia in DM patients.^10^ Anaemia represents an emerging global health problem that negatively impacts the quality of life and requires an ever‐greater allocation of healthcare resources. It also induces reduced exercise capacity, fatigue, anorexia, depression, cognitive dysfunction, and decreased libido that increase the risk of cardiac disease and depress the life expectancy of patients. Anaemia is found to contribute to the development and progression of micro‐and macro‐vascular complications in DM patients.^11^ It is associated with a rapid decline of renal function and an increased need for renal replacement therapy, which is often unavailable or unaffordable in most developing countries like Ethiopia.^12^ People who have both DM and anaemia are more likely to die early than those who have DM but not anaemia.^13^

Under these circumstances, anaemia in patients with DM must be treated once diagnosed, since it may contribute to the pathogenesis and progression of cardiovascular disease and serious diabetic nephropathy and retinopathy. The regular screening for anaemia along with other DM‐associated complications can help slow the progression of vascular complications in these patients.^14^

To the best of our knowledge, this is the first systematic review and meta‐analysis to summarize all available data on the prevalence of anaemia in DM patients in Ethiopia. There is also no official nationwide survey or national health registry that has to date estimated the prevalence of anaemia in DM patients in the country. Therefore, the main aim of this study is to measure the countrywide pooled prevalence and associated factors of anaemia in DM patients.

## 2 METHODS

### 2.1 Design and protocol registration

This systematic review and meta‐analysis was conducted as per the PRISMA guideline^15^ (Table S1). The protocol has been registered in the International Prospective Register of Systematic Reviews (PROSPERO), with the registration number of CRD42021225549.

### 2.2 Study setting

This is a systematic review and meta‐analysis of published articles on the prevalence and associated factors of anaemia among DM patients in Ethiopia.

### 2.3 Search strategy

A review of all published articles was done in the following major databases: PubMed, Cochrane Library, Google Scholar, and African Journals Online. The search for published studies was not restricted by time, and all published articles up to February 2021 were included in this review. It was aided with manual searches to identify relevant unpublished studies. The reference lists of retrieved articles were searched to identify any studies that are not retrieved from electronic databases. The search terms were organized following the Medical Subject Headings thesaurus (MESH) using the following terms, “anemia”, “hematological parameters”, “red blood cell parameters”, “diabetes mellitus”, “determinant factors of anemia”, “associated factors of anemia” and “Ethiopia” (Table S3).

### 2.4 Eligibility criteria

All cross‐sectional studies which reported the prevalence of anaemia among DM patients in Ethiopia using the English language and published in peer‐reviewed journal were included. Studies such as review articles, abstracts, editorials and case controls were excluded from this study. Articles that did not report specific outcomes for anaemia and associated factors were also excluded from this systematic review and meta‐analysis.

### 2.5 Outcomes of the study

The main outcome of interest was the prevalence of anaemia per the World Health Organization (WHO) definition for anaemia (haemoglobin (Hgb) value less than 12 g/dl for men and less than 11 g/dl for women). ^16^ The secondary outcome of this study was assessing the factors associated with the prevalence of anaemia in DM patients in Ethiopia.

### 2.6 Study selection and quality appraisal

All articles retrieved through search strategy were imported to EndNote X7 (Thomson Reuters). After excluding duplicated articles, titles/abstracts were independently screened by two review authors (TA and SG). Possible arguments between two review authors were solved through discussions and mutual consensus. Whenever further information is required, we made contact with the author by email. Quality assessment was conducted based on JBI critical appraisal checklist for simple prevalence using 9 criteria.^17^ The checklist consists of nine items: (1) Was the sample frame appropriate to address the target population? (2) Were study participants sampled appropriately? (3) Was the sample size adequate? (4) Were the study subjects and the setting described in detail? (5) Was the data analysis conducted with sufficient coverage of the identified sample? (6) Were valid methods used for the identification of the condition? (7) Was the condition measured in a standard, reliable way for all participants? (8) Was there an appropriate statistical analysis? (9) Was the response rate adequate? For each question, a score was assigned (0 for ‘not reported or not appropriate’ and 1 for ‘yes’); the scores were summarized across the items to get a total quality score that ranged from 0 to 9. Studies were then classified as having a low, medium and high quality when the awarded points become 0–4, 5–7 and 7–9, respectively. Articles having high and medium quality were included in the final analysis (Table S2).

### 2.7 Data extraction

Data extracted from relevant studies were summarized into an excel spreadsheet. Data extraction sheet included study characteristics such as (1) Authors’ name, year of study, study setting, publication year, study design, sampling techniques, duration of the disease (DM), and the number of anaemic patients (cases).

### 2.8 Data analysis

Relevant data were entered into Microsoft Excel and then exported to STATA version 11 (STATA Corp LLC) for further analysis. The pooled prevalence of anaemia among DM patients was conducted using a random effects model along with 95% CI. Index of heterogeneity (*I* ^2^ statistics) was used to quantitatively measure the magnitude of heterogeneity among the included studies. Low, medium, and high heterogeneity was considered when the values of *I* ^2^ is 25%, 50%, and 75%, respectively.^18^ A subgroup and sensitivity analysis were conducted to determine the potential sources of heterogeneity among the included studies. Funnel plot analysis and Egger weighted regression tests were conducted to detect publication bias. A *p*‐value < .05 in the Eggers test was considered as evidence of publication bias.^19^ The effect size of the included studies was extracted, and the pooled effect size was determined by analysis. The effect size of categorical data was expressed using OR.

## 3 RESULT

### 3.1 Description of studies

**FIGURE 1:**
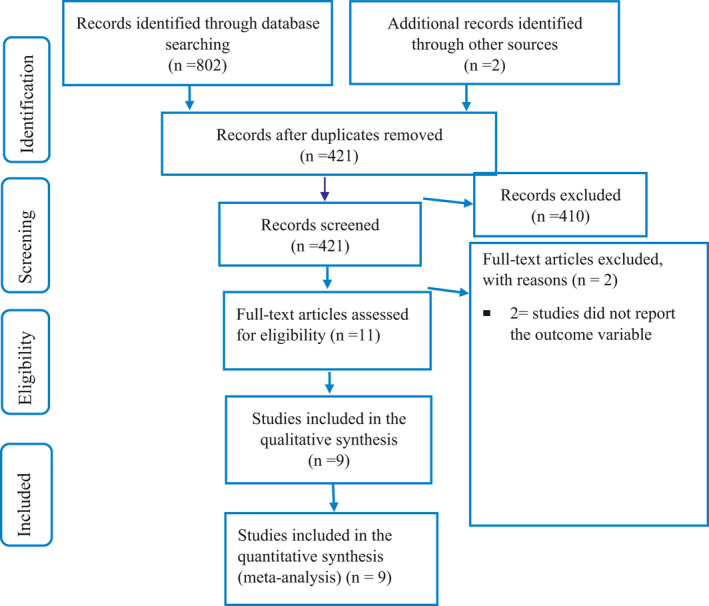
Flow chart to describe the selection of studies for the systematic review and meta‐analysis on the prevalence of anaemia among DM patients in Ethiopia

A total of 802 articles were retrieved by the literature search. Of these, 383 were excluded due to duplication, 2 did not relate to the aim of this study, and finally, 9 studies were included in this systematic review and meta‐analysis (Figure 1).

### 3.2 Characteristics of included studies

Nine full‐text articles were included in this systematic review and meta‐analysis. All articles followed a cross‐sectional study design. The number of DM patients as per studies ranged from 246 in Gondar^20^ to 412 in Dessie,^21^ with a total of 2889 patients across all studies. The included articles were published between 2012 and 2021. Three regional states (provinces) of Ethiopia were represented in the studies: six studies were conducted in Amhara,^20^, ^21^, ^22^, ^23^, ^24^, ^25^ 1 study in Tigray,^26^ and 2 study in Oromo regional state.^27^ Six studies^21^, ^22^, ^23^, ^24^, ^26^, ^28^ had used a systematic sampling strategy while the other 3 studies^20^, ^27^ used a simple random sampling to select their representative sample. The mean age of study participants ranged from 40 to 56.3 years. The average duration of illness among DM patients was reported in 5 studies, ranging from 4 to 8.87 years (Table 1).

**TABLE 1:** Descriptive summary of 9 included studies on the prevalence of anaemia in DM patients in Ethiopia

Author, year of publication	Study setting	Study year	Study design	Sample size	Sampling method	Cases	Mean age (years)	DM duration (years)	Anaemia (gender)		Prevalence (%)
									Male	Female	
Adane et al., 2020^20^	Gondar	2019	Cross‐sectional	246	Simple random	22	50	6.0	81	19	13.4
Fiseha et al., 2019^21^	Dessie	2018	Cross‐sectional	412	Systematic	110	45 ± 14.6	4	35.5	19.7	26.7
Abate et al., 2013^22^	Fenote selam	2012	Cross‐sectional	384	Systematic	73	40.96 ± 16.8	5.87 ± 0.47	52.1	47.9	19
Kebede et al., 2021^23^	Gondar	2019	Cross‐sectional	372	Systematic	30		8.87 ± 3.69	10.56	6.52	8.06
Taderegew et al., 2020^24^	Debre‐birhan	2019	Cross‐sectional	249	Systematic	50	53.71 ± 10.41	7.49 ± 4.6	19.01	21.1	20.1
Engdaw et al., 2020^25^	Debre‐tabor	2019	Cross‐sectional	265	Simple random	79	48.69 ± 15.92	–	43	57	29.8
Hailu et al., 2020^26^	Tigray	2019	Cross‐sectional	262	Systematic	47	NR	8.7 ± 6.8	12.7	22.4	17.9
Bekele et al., 2019^27^	Harari	2019	Cross‐sectional	374	Simple random	130	56.3 ± 11.5	5.0	41.5	28.8	34.8
Tujuba et al., 2021^28^	West hararge	2020	Cross‐sectional	325	Systematic		40	4.5 ± 4.0	36	20.5	30.2

### 3.3 Prevalence of anaemia

**FIGURE 2:**
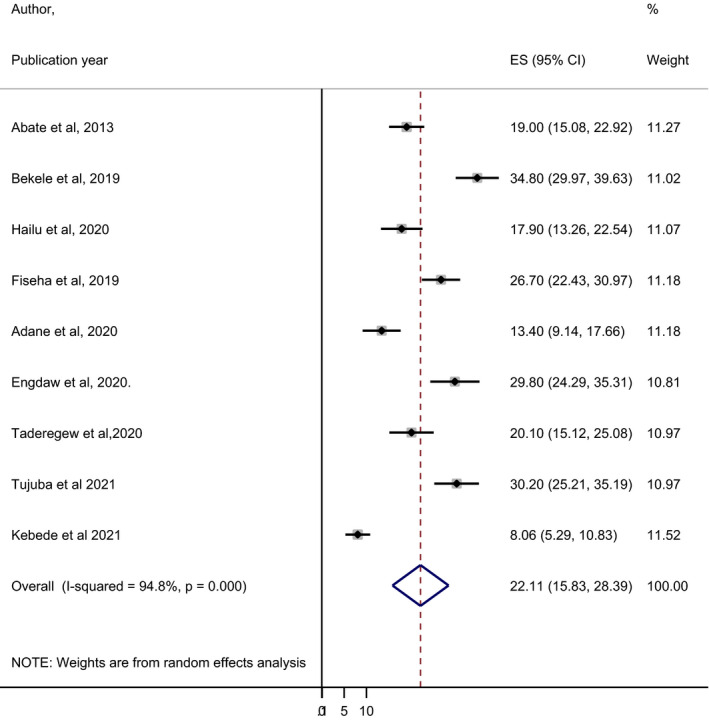
Forest plot displaying the pooled prevalence of anaemia among DM patients

Nine published studies were included in this systematic review and meta‐analysis to estimate the pooled prevalence of anaemia in DM patients. The minimum and maximum prevalence of anaemia were 8.06% and 34.8% in Gondar^23^ and Harari,^27^ respectively. The overall pooled prevalence of anaemia in DM patients using the random effects model was 22.11% (95% CI: 15.83–28.39) *I* ^2^ = 94.8%. Using *I* ^2^ statistics, we have assessed the heterogeneity of the included studies and it was significant (*I* ^2^ = 94.8%, *p* < .001), showing a high level of heterogeneity among the included studies. The statistical analyses of the prevalence of anaemia in DM patients are presented in Figure 2.

### 3.4 Subgroup analysis

Since there is a high level of heterogeneity in this review, subgroup analysis was done by considering the type of DM, gender, study setting, and duration of illness as a grouping variable. Accordingly, the prevalence of anaemia was higher in type II DM patients (31.12% [95% CI; 9.66–52.58]) than in type I DM patients (16.78% [95% CI: 11.53–22.04]). The pooled prevalence of anaemia was 36.72% (95% CI: 22.58–50.87) *I* ^2^ = 97.6% and 27.51% (95% CI: 16.12–38.90) *I* ^2^ = 96.3% among male and female, respectively. The combined prevalence of anaemia among Amhara regions and other regions was 19.41% (95% CI: 13.06–25.76) *I* ^2^ = 86.7% and 26.34% (95% CI: 9.77–42.90) *I* ^2^ = 95.9%, respectively. The results of subgroup analysis indicated that the source of heterogeneity might be due to the type of DM, gender, study setting, and duration of illness. The subgroup analysis of the prevalence of anaemia in DM patients is presented in Table 2.

**TABLE 2:** Subgroup analysis describing the pooled prevalence of anaemia among DM patients in Ethiopia

Subgroup	Included studies	Prevalence (95% CI)	Heterogeneity statistics	*p*‐Value	*I* ^2^
Gender					
Male	9	36.72 (22.58 −50.87)	331.26	˂.001	97.6
Female	9	27.51 (16.12–38.90)	189.87	˂.001	96.3
Study setting					
Amhara region	6	19.41 (13.06–25.76)	61.33	˂.001	86.7
Other regions	3	27.61 (17.52–37.70)	26.32	˂.001	92.4
Duration of illness					
˂6 years	4	27.57 (20.80–34.34)	27.49	˂.001	89.1
≥6 years	5	17.70 (10.88–24.53)	43.15	˂.001	90.7
Type of DM					
Type I	3	16.78 (11.53–22.04)	10.77	.005	81.4
Type II	6	35.75 (12.37–59.14)	817.11	˂.001	99.4

### 3.5 Association between anaemia, age, duration of illness, eGFR and glycemic control

**FIGURE 3:**
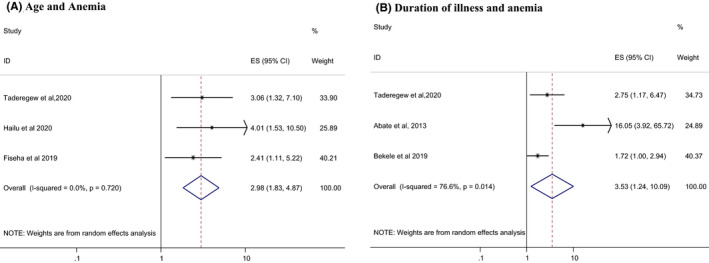
Forest plots which describe associated factors (age and duration of illness) of anaemia among DM patients in Ethiopia

**FIGURE 4:**
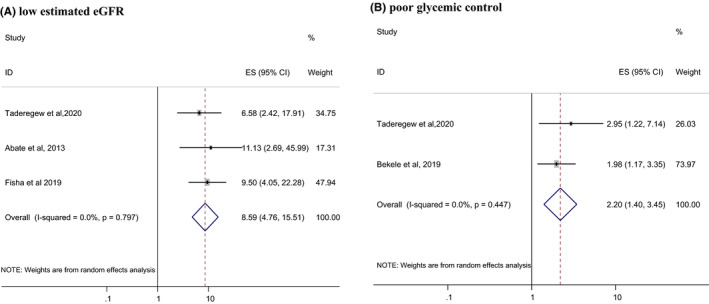
Forest plots which describe associated factors (low eGFR and poor glycemic control) of anaemia among DM patients in Ethiopia

Low estimated glomerular filtration rate (eGFR) (OR = 8.59; 95% CI: 4.76, 15.57), duration of illness for more than 5 years (OR = 2.66; 95%: 1.38, 5.13), poor glycemic control (OR = 2.20; 95% CI: 1.40, 3.45), and age ˃ 60 years (OR = 2.98; 95% CI: 1.83, 4.87) were found to be independent predictors for the occurrence of anaemia among DM patients (Figures 3 and 4).

### 3.6 Publication bias

**FIGURE 5:**
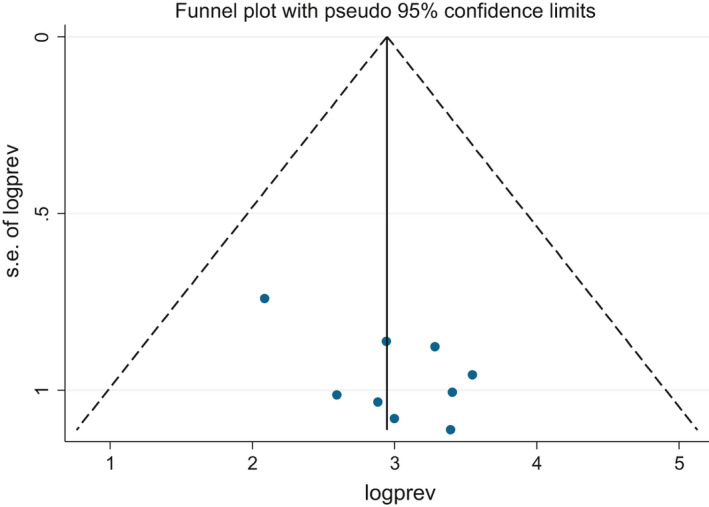
Funnel plot of included studies on the prevalence of anaemia among DM patients

We used a Funnel plot and Egger's test to check the presence of publication bias. The Funnel plot is symmetrical (Figure 5), and Egger's test result was 16.26 (95% CI: 6.45, 26.06, *p*‐value = .006) (Table 3), indicating that there is no publication bias among the included studies.

**TABLE 3:** Egger's test

	Standard effect	Coefficient	Standard error	*p *> |*t*|	[95% Confidence interval]	
Slope	−14.80526	8.901983	−1.66	.140	−35.8551	6.244586
Bias	16.26184	4.146862	3.92	.006	6.456065	26.06761

### 3.7 Sensitivity analysis

Due to the high heterogeneity of results, a sensitivity analysis was done by applying a random effects model. The analysis was done to evaluate the effect of each study on the pooled prevalence of anaemia by excluding each study step‐by‐step. Its results showed that the omitted studies did not have a significant effect on the pooled prevalence of anaemia among DM patients (Table 4).

**TABLE 4:** Sensitivity analysis of the included studies to estimate the prevalence of anaemia among DM patients

Study omitted	Estimate (95% CI)	Heterogeneity	
		*I* ^2^	*p*‐value
Adane et al. 2020^20^	24.64 (19.27, 30.02)	87.6%	˂.001
Fiseha et al. 2019^21^	22.41 (16.08, 28.74)	91.1%	˂.001
Abate et al. 2013^22^	23.73 (17.23, 30.23)	91.2%	˂.001
Kebede et al. 2021^23^	23.91 (18.77–29.04)	89.9	˂.001
Taderegew et al. 2020^24^	23.51 (17.14, 29.89)	91.6%	˂.001
Engdaw et al. 2020^25^	21.94 (16.03, 27.86)	90.6%	˂.001
Hailu et al. 2020^26^	23.89(17.61, 30.16)	91.2%	˂.001
Bekele et al. 2019^27^	21.04 (16.38, 25.69)	84.1%	˂.001
Tujuba et al. 2021^28^	23.02 (17.52, 28.52)	90.1%	˂.001
Combined	22.11 (15.83,28.39)	94.8%	˂.001

### 3.8 Meta‐regression

Meta‐regression was performed on continuous covariates such as study year, the mean age of the participants, sample size, and duration of illness. Accordingly, the result of the meta‐regression showed that the pooled prevalence of anaemia among DM patients was not associated with the above listed variables (Table 5).

**TABLE 5:** Meta‐Regression of factors associated with heterogeneity in this study

Variables	Coefficient	*p*‐Value
Mean age	−0.035	.574
Study year	−0.267	.066
Sample size	0.001	.814
Duration of illness	0.180	.594

## 4 DISCUSSION

Anaemia is a global public health problem affecting both developing and developed countries with major consequences for human health as well as social and economic development.^29^ Nine articles on a total of 2889 DM patients were included in this systematic review and meta‐analysis to estimate the pooled prevalence and associated factors of anaemia among DM patients in Ethiopia. The overall pooled prevalence of anaemia was 22.11% (95% CI: 15.83–228.39) *I* ^2^ = 94.8%. The result of this review revealed that one in five DM patients was found to be anaemic in Ethiopia. As per the definition of WHO, anaemia is considered a moderate public health problem when the prevalence exceeds 20% of the population.^16^ Accordingly, the result of this review implies that anaemia is a moderate public health problem among DM patients in Ethiopia, which needs to design prevention and control strategies to reduce the burden of anaemia.

In subgroup analysis, the prevalence of anaemia was 16.78% (95% CI: 11.53–22.04) and 31.12% (95% CI; 9.66–26.06) in type I and type II DM patients, respectively. This finding was in corroborating with the notion that anaemia is a more frequent condition in type II DM patients.^30^ Type II DM patients are more vulnerable to various forms of both short‐term and long‐term complications due to the commonness of this type of DM, its insidious onset and late recognition, especially in developing countries.^31^ Hyperglycaemia in type II DM has a direct effect on the development of inflammatory conditions induced by the increased expression of proinflammatory cytokines such as interleukin‐6 (IL‐6). The high level of IL‐6 causes antierythropoietic effect, changes the sensitivity of progenitor cells to Epo and promotes apoptosis of immature RBCs causing to development of anaemia.^32^, ^33^

The result of the subgroup analysis showed a lower prevalence of anaemia in the Amhara region (19.41%) than in other regions (26.34%). The difference in the prevalence of anaemia across provinces in Ethiopia might be attributed because of the difference in geographical altitude and the number of studies included in each category of analysis. The results by gender also showed that the prevalence of anaemia was higher in men (36.72%) than in women (27.51%). This might be explained by the fact that male patients with DM are commonly affected by low testosterone levels and hypo‐gonadotropic hypogonadism. Since testosterone stimulates the production of RBCs, low testosterone levels may contribute to anaemia in male patients.^34^

In the random effect model, the pooled effect size of anaemia among aged patients (˃60 years) was 2.98 (OR = 2.98; 95% CI: 1.83, 4.87) times higher when compared with those of age ˂60 years. This finding is consistent with the findings reported in the study conducted in Nigeria^35^ and Australia.^36^ The possible reasons for this increased prevalence with age might be due to deficiencies of vitamins such as folate, cyanocobalamin or bone marrow disorders and a higher number of comorbidities.^37^ Anaemia is a common clinical problem at all ages, but this is especially true among the elderly.^38^ The prevalence of anaemia increases with increasing age, affecting approximately 10% of the general population ≥65 years of age. However, the prevalence of anaemia in patients with DM could be double this figure.^36^ Although it is anticipated that a decrease in the Hgb level might be a normal consequence of ageing (irrespective of their health status), evidence has suggested that anaemia does reflect poor health and increased vulnerability to adverse outcomes in older persons.^22^ Many underlying diseases develop preferentially in elderly individuals. At least one‐third of anaemic patients older than 65 years show a hyper‐inflammatory state typical for chronic kidney disease (CKD) or autoimmune disease and chronic infection.^39^

In this meta‐analysis, the odds of developing anaemia were 2.66 (OR = 2.66; 95%: 1.38, 5.13) times higher among DM patients with a duration of illness of ≥5 years compared with DM patients with a duration of illness of <5 years. This finding is in corroborating with other studies conducted in Nigeria^40^ and India.^41^ The reason for this increased risk of being anaemic with increasing duration of DM may be due to the effects of DM‐related chronic hyperglycaemia. This is not surprising as longer exposure to hyperglycaemia and target organ damage likely puts patients at higher risk of complications including anaemia.^40^ These observations suggest that anaemia evaluation should be considered in the routine management of persons with DM and should be treated to minimize the risk of microvascular complications.^42^

The odds of developing anaemia were 8.59 times higher among DM patients having low eGFR (˂60 ml/min) compared to their counterparts (OR = 8.59; 95% CI: 4.76, 15.57). This finding is supported by another studies conducted in Australia^43^ and California.^44^ Patients with DM and renal insufficiency have a higher risk of developing anaemia associated with decreased production of Epo due to kidney failure. It has been also suggested that DM patients are more vulnerable to a significant additional burden to anaemia in the presence of renal insufficiency.^45^ Generally, anaemia is more frequent and severe in DM patients at any level of GFR compared to non‐diabetic patients.^42^

On the other hand, patients with poor glycemic control were 2.20 times (OR = 2.20; 95% CI: 1.40, 3.45) more likely to be anaemic compared to their counterparts. This finding is in line with studies conducted in Kuwait^46^ and Pakistan.^47^ In patients with poorly controlled diabetes, the RBC precursors of the bone marrow might be prone to prolonged direct toxicity to glucose toxicity or the mature RBCs can be affected by oxidative stress leading to disturbances in the RBCs function.^48^ DM‐related autonomic neuropathy is a major complication of poor glycemic control. Since Epo production and release is regulated by the autonomic nervous system, therefore higher incidence of anaemia in poorly controlled DM patients is due to impaired Epo production.^35^, ^49^ Other factors increasing the risk of anaemia include systemic inflammation; damage to renal architecture produced by chronic hyperglycaemia and consequent formation of advanced glycation end‐products; and depressed androgen levels induced by DM. It is speculated that these conditions may be aggravated in poorly controlled DM than in controlled DM.^35^

### 4.1 Strengths and limitations

To the best of our knowledge, this is the first systematic review and meta‐analysis to summarize all the available data on the prevalence of anaemia among DM patients in Ethiopia. The information provided in this study may play a positive role in improving public health interventions in the country, as there is no national registry to determine the pooled prevalence of anaemia in DM patients. In this review, we used a comprehensive search strategy and more than one reviewer was involved in each step of the review process. PRISMA guideline was strictly followed during the whole review process. The result of this study should be considered with some limitations in mind: the levels of heterogeneity between included studies were high, which can be attributed to variation in sample size, study period and geographic location.

## 5 CONCLUSION

The result of this review implies that anaemia is a moderate public health problem among DM patients in Ethiopia. Older age, poor glycemic control, low eGFR, and longer duration of illness were found to be the contributing factors for the development of anaemia in DM patients. Therefore, by considering the negative impact of anaemia, it is important to include anaemia screening into routine assessment of DM‐related complications targeting patients with older age, poor glycemic control, low eGFR​, and longer duration of illness to reduce the magnitude of the problem.

## CONFLICTS OF INTEREST

The authors declare that they have no competing interests.

## AUTHOR CONTRIBUTION

TA‐conceptualization, literature search, writing original draft, statistical analysis and quality assessment. SG‐conceptualization, literature search, statistical analysis and review and editing. All the authors critically revised the paper and agreed to be accountable for all aspects of the work.

## ETHICAL APPROVAL AND CONSENT TO PARTICIPATE

Not applicable.

## Supporting information

Table S1

Table S2

Table S3

## Data Availability

The data sets used and/or analysed during the current study are available from the corresponding author on reasonable request.
