# Supplementary material for: Anaemia and its associated factors among diabetes mellitus patients in Ethiopia: A systematic review and meta‐analysis
Source: Endocrinol Diabetes Metab. 2021 May 14;4(3):e00260. doi: 10.1002/edm2.260 (PMC8279623; doi:10.1002/edm2.260)
Supplement: Supplementary file 1 — Table S1 [file EDM2-4-e00260-s001.docx]

| Author, year of publication | Q1 | Q2 | Q3 | Q4 | Q5 | Q6 | Q7 | Q8 | Q9 | Total score (9%) |
| --- | --- | --- | --- | --- | --- | --- | --- | --- | --- | --- |
| Adane et al, 2020 ^20^ | Y | N | Y | Y | Y | Y | Y | Y | Y | 8 |
| Fiseha et al, 2019 ^21^ | Y | Y | Y | Y | Y | Y | Y | Y | Y | 9 |
| Abate et al, 2013 ^22^ | Y | Y | Y | Y | Y | Y | Y | Y | NR | 8 |
| Taderegew et al, 2020 ^24^ | N | Y | Y | Y | Y | Y | Y | Y | NR | 8 |
| Hailu et al, 2020 ^26^ | Y | Y | Y | Y | Y | Y | Y | Y | NR | 8 |
| Bekele et al, 2019 ^27^ | Y | N | Y | Y | Y | Y | Y | Y | Y | 8 |
| Engdaw et al, 2020 ^25^ | Y | Y | Y | Y | NR | NR | Y | Y | Y | 7 |
| Tujuba et al 2021^28^ | Y | Y | Y | Y | NR | Y | Y | Y | NR | 7 |
| Kebede et al 2021 ^23^ | Y | Y | Y | NA | Y | Y | NA | Y | Y | 7 |

Table S2 Quality assessment of the studies included in systematic review and meta-analysis of anemia and its associated factors among diabetes mellitus patients in Ethiopia

**Key:** **Y**= Yes; **NR**= Not reported, **NA**=Not appropriate

**Question codes:**

1. Was the sample frame appropriate to address the target population?

2. Were study participants sampled in an appropriate way?

3. Was the sample size adequate?

4. Were the study subjects and the setting described in detail?

5. Was the data analysis conducted with sufficient coverage of the identified sample?

6. Were valid methods used for the identification of the condition?

7. Was the condition measured in a standard, reliable way for all participants?

8. Was there appropriate statistical analysis?

9. was the response rate adequate, and if not, was the low response rate managed appropriately?
