# Supplementary material for: Anaemia and its associated factors among diabetes mellitus patients in Ethiopia: A systematic review and meta‐analysis
Source: Endocrinol Diabetes Metab. 2021 May 14;4(3):e00260. doi: 10.1002/edm2.260 (PMC8279623; doi:10.1002/edm2.260)
Supplement: Supplementary file 3 — Table S3 [file EDM2-4-e00260-s002.docx]

Table S Search strategy for PubMed

| Search | Search terms | Hits |
| --- | --- | --- |
| #1 | ((((anemia[Text Word]) OR (hematological parameters[Text Word])) OR (hematological profile[Text Word])) OR (red cell parameters[Text Word])) OR (anaemia[Text Word]) | 205,519 |
| #2 | ((((anemia[MeSH Terms]) OR (hematological parameters[MeSH Terms])) OR (hematological profile[MeSH Terms])) OR (red cell parameters[MeSH Terms])) OR (anaemia[MeSH Terms]) | 164,020 |
| #3 | (((((anemia[Text Word]) OR (hematological parameters[Text Word])) OR (hematological profile[Text Word])) OR (red cell parameters[Text Word])) OR (anaemia[Text Word])) OR (((((anemia[MeSH Terms]) OR (hematological parameters[MeSH Terms])) OR (hematological profile[MeSH Terms])) OR (red cell parameters[MeSH Terms])) OR (anaemia[MeSH Terms])) | 236,623 |
| #4 | ((diabetics patients[Text Word]) OR (DM[Text Word])) OR (Diabetes mellitus[Text Word]) | 482,751 |
| #5 | ((((((anemia[Text Word]) OR (hematological parameters[Text Word])) OR (hematological profile[Text Word])) OR (red cell parameters[Text Word])) OR (anaemia[Text Word])) OR (((((anemia[MeSH Terms]) OR (hematological parameters[MeSH Terms])) OR (hematological profile[MeSH Terms])) OR (red cell parameters[MeSH Terms])) OR (anaemia[MeSH Terms]))) AND (((diabetics patients[Text Word]) OR (DM[Text Word])) OR (Diabetes mellitus[Text Word])) | 3947 |
| #6 | Ethiopia[Text Word] | 20,914 |
| #7 | (((((((anemia[Text Word]) OR (hematological parameters[Text Word])) OR (hematological profile[Text Word])) OR (red cell parameters[Text Word])) OR (anaemia[Text Word])) OR (((((anemia[MeSH Terms]) OR (hematological parameters[MeSH Terms])) OR (hematological profile[MeSH Terms])) OR (red cell parameters[MeSH Terms])) OR (anaemia[MeSH Terms]))) AND (((diabetics patients[Text Word]) OR (DM[Text Word])) OR (Diabetes mellitus[Text Word]))) AND (Ethiopia[Text Word]) | 10 |
